# Gender Identification of Human Cortical 3-D Morphology Using Hierarchical Sparsity

**DOI:** 10.3389/fnhum.2019.00029

**Published:** 2019-02-07

**Authors:** Zhiguo Luo, Chenping Hou, Lubin Wang, Dewen Hu

**Affiliations:** ^1^College of Mechatronics and Automation, National University of Defense Technology, Changsha, China; ^2^College of Science, National University of Defense Technology, Changsha, China; ^3^Cognitive and Mental Health Research Center, Beijing Institute of Basic Medical Science, Beijing, China

**Keywords:** cortical three-dimensional morphology, gender difference, hierarchical sparse representation classifier, Magnetic Resonance Imaging, multivariate pattern analysis

## Abstract

Difference exists widely in cognition, behavior and psychopathology between males and females, while the underlying neurobiology is still unclear. As brain structure is the fundament of its function, getting insight into structural brain may help us to better understand the functional mechanism of gender difference. Previous structural studies of gender difference in Magnetic Resonance Imaging (MRI) usually focused on gray matter (GM) concentration and structural connectivity (SC), leaving cortical morphology not characterized properly. In this study a large dataset is used to explore whether cortical three-dimensional (3-D) morphology can offer enough discriminative morphological features to effectively identify gender. Data of all available healthy controls (*N* = 1113) from the Human Connectome Project (HCP) were utilized. We suggested a multivariate pattern analysis method called Hierarchical Sparse Representation Classifier (HSRC) and got an accuracy of 96.77% for gender identification. Permutation tests were used to testify the reliability of gender discrimination (*p* < 0.001). Cortical 3-D morphological features within the frontal lobe were found the most important contributors to gender difference of human brain morphology. Moreover, we investigated gender discriminative ability of cortical 3-D morphology in predefined Anatomical Automatic Labeling (AAL) and Resting-State Networks (RSN) templates, and found the superior frontal gyrus the most discriminative in AAL and the default mode network the most discriminative in RSN. Gender difference of surface-based morphology was also discussed. The frontal lobe, as well as the default mode network, was widely reported of gender difference in previous structural and functional MRI studies, which suggested that morphology indeed affect human brain function. Our study indicates that gender can be identified on individual level by using cortical 3-D morphology and offers a new approach for structural MRI research, as well as highlights the importance of gender balance in brain imaging studies.

## 1. Introduction

Gender difference has been widely reported in psychiatric and neurological diseases (Piccinelli and Wilkinson, 2000; Baron-Cohen et al., 2005; Shulman, 2007; Eranti et al., 2013; Lai et al., 2015), cognitive functions (Ren et al., 2009; Ohla and Lundstr, 2013; Yin et al., 2017; Chen et al., 2018) and behaviors (Christov-Moore et al., 2014), while its neurobiological mechanism is unclear yet (Giudice, 2009). As neural function has its structural basis, studying brain neuroanatomy may provide us new insights and understandings of gender difference.

Previous reports tend to explain gender difference in the view of GM concentration, SC and Functional Connectivity (FC). Wang et al. (2012) applied multivariate pattern analysis on GM concentration and resting state fMRI from healthy young adults and got an accuracy of 89%, and they found the occipital lobe and the cerebellum the most discriminative regions of gender difference; Yuan et al. (2018a) proposed a three-dimensional weighted histogram of gradient orientation to describe the complex spatial structure of human brain image, and they got an over 90% accuracy of gender classification on 527 healthy adults from four research sites; Ruigrok et al. (2014) reported gender difference in the amygdala, hippocampus, and insula after meta-analysis in human brain structure; Goldstein et al. (2001) found females had higher percentage of GM than males, while Gur et al. (1999) got a converse result in white matter; Feis et al. (2013) used multimodal gender classification of T1-weighted, T2-weighted and fractional anisotropy images and indicated the frontal lobe the most discriminative lobe. Gong et al. (2009) found greater overall cortical connectivity and more efficient cortical network organizations in women; Ingalhalikar et al. (2013) reported that males had stronger intra-hemispheric SC while females had stronger inter-hemispheric SC using diffusion tensor imaging. Zhang et al. (2018) used 4 fMRI runs of 820 healthy controls from the HCP and got the accuracy of 87% using FC features for gender prediction, and they suggested that FC within the default, fronto-parietal and sensorimotor networks had the greatest gender prediction abilities while the right fusiform gyrus and the right ventromedial prefrontal cortex contributed the most in the default mode network.

Recently, gender difference in surface-based morphology such as cortical thickness, surface area, cortical curvature and cortical volume has attracted much attention. Im et al. (2006) indicated that women showed more significant localized cortical thickening in the frontal, parietal and occipital lobes, which were also reported of significant gender-related difference by Lv et al. (2010) using graph theoretical approaches; Sowell et al. (2007) found women had thicker cortices in posterior temporal and right inferior parietal regions, while men showed larger brain in all locations, especially in the frontal and occipital poles of both hemispheres; Sepehrband et al. (2018) developed a multivariate statistical learning model to predict gender from regional neuroanatomical features on different brain atlases, and they got an 83% cross-validated prediction accuracy and found the middle occipital lobes and the angular gyri the major predictors of gender.

Despite studies of gender difference in surface-based morphology, few paid attention to the original cortical 3-D morphology, which is defined as the voxel-based morphology of the cerebral cortex without gray matter concentration in the standard MNI space. Clearly the original cortical 3-D morphology contains more abundant and complete morphological information, and most surface-based morphology such as cortical thickness and curvature are measured on the cortical 3-D morphology (cortical volume and surface area are measured in the subject's undistorted native volume space). Moreover, most previous morphology studies focused on finding gender difference using statistical analysis while few of them have effectively discriminated males from females with high classification accuracy using those morphological features to support their conclusions.

In this study, we aimed to find gender difference of cortical 3-D morphology and focused on two questions: (a) Can gender be discriminated with a high accuracy using cortical 3-D morphology? (b) What is the most discriminative region of gender in cortical 3-D morphology?

## 2. Materials and Methods

### 2.1. Data Acquisition and Preprocessing

Structural MRI was acquired from the HCP S1200 release, and details about the HCP can be seen in Essen et al. (2012). Subjects were scanned on a customized 3T Siemens scanner (Connectome Skyra) with a standard 32-channel head coil and a body transmission coil and scan parameters were as follows: TR = 2400 ms, TE = 2.14 ms, Voxel Size = 0.7 mm isotropic. All 1113 available subjects (age: 22–37 years, gender: 507 males and 606 females) were selected for our gender difference study.

**Figure 1:**
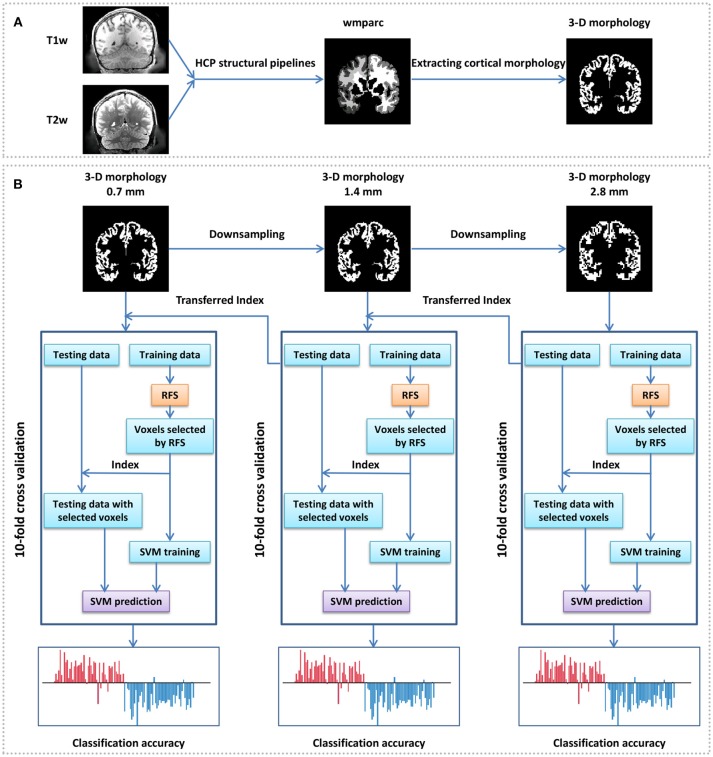
Framework of gender identification of cortical 3-D morphology via HSRC. **(A)** Process of cortical 3-D morphology extraction. For each subject, T1w and T2w were used in the HCP structural pipelines to generate a normalized volume parcellation—the wmparc, which is an accurate subject-specific human brain mask of the gray matter and white matter in the MNI space. We defined the value of the gray matter voxels as 1 and others as 0, and got the original cortical 3-D morphology. **(B)** Gender classification with cortical 3-D morphology using HSRC. The original cortical 3-D morphology (0.7 mm) of each subject was first downsampled into 1.4 and 2.8 mm, then gender classification was conducted on the 2.8 mm 3-D morphology with 10-fold cross validation, RFS was used on the training data to select voxels in each fold. We set the overall classification accuracy as a function of the number of selected voxels in each fold, and selected the union of the selected voxels in each fold corresponding to the highest accuracy as discriminative voxels, the corresponding voxels in 1.4 mm morphology were selected as the initial input for the next 10-fold across validation. The same operation was conducted in 0.7 mm data.

Data were initially preprocessed by the HCP structural pipelines in this study, and a highlight of the HCP pipelines is that it uses T2-weighted structural images for registration so as to get more precise registration and segmentation results. The main preprocessing steps include gradient distortion correction, brain extracting, readout distortion correction, boundarybased cross-modal registration, bias field correction, recon-all pipeline in FreeSurfer, and native to MNI nonlinear volume transformation, and detailed preprocessing steps can be seen in Glasser et al. (2013). One of the outputs, the wmparc, is an accurate subject-specific human brain mask of the gray matter and white matter in the MNI space. In the file “MNINonLinear/wmparc.nii” of each subject of the HCP, the scattered integers between 251 and 2035 stand for different subregions of the cerebral cortex, and when they were defined as 1 and others as 0, the original 3-D morphology of the cerebral cortex were obtained (Figure 1A). We also attempted to analyse the discriminative abilities of both anatomical and functional subregions, so atlas-based morphology analysis (Meyer et al., 2017) was conducted with two predefined atlas: the AAL template (Tzourio-Mazoyer et al., 2002) was used as structural atlas and the 7 RSN template (Thomas Yeo et al., 2011) was used as functional atlas (https://surfer.nmr.mgh.harvard.edu/fswiki/CorticalParcellation_Yeo2011, “Yeo2011_7Networks_MNI152_FreeSurfer-Conformed1mm_LiberalMask.nii,” downsampled to 1.4 mm isotropic). All the MRI files and templates were in the standard MNI space for comparisons across subjects.

As surface-based morphology was discussed in this study, we obtained 4 surface-based morphological features (thickness, curvature, sulc and myelinmap) in the HCP for gender difference analysis. They were all spatially downsampled to a ~32k mesh of each hemisphere (average vertex spacing of ~2 mm).

### 2.2. Hierarchical Sparsity Feature Selection

Considering the scale of the dataset in this study, a 10-fold cross validation was conducted for gender classification, and in consideration of numerous features of MRI data (dimensionality=1,113 × 4,352,560 after abandon all-0 and all-1 columns for 0.7 mm data matrix), dimensionality reduction is essential to alleviate or avoid the curse of dimensionality (Liu and Motoda, 1998).

Feature extraction algorithms like Principal Component Analysis (PCA) combine all features to create new dimensionality reduced features in a new feature space, and general statistical tests like *t*-test are unsuitable to filter 0-1 distributed features. Comparatively, sparse representations select typical features from the original feature space directly, so that we can maintain the original physical meanings of the cortical morphological features and have a better explanation.

Since sparse representation is not good at dealing with data with too large dimensionality (Su et al., 2012), we proposed a Hierarchical Sparse Representation Classifier (HSRC) algorithm for informative feature selection and classification (Figure 1B). MRI data were downsampled to voxel size=1.4 mm isotropic (feature dimensionality=544,069 after abandon all-0 and all-1 columns) and voxel size=2.8 mm isotropic (feature dimensionality=67,994 after abandon all-0 and all-1 columns). The 10-fold cross-validation classification was first conducted in 2.8 mm data. In each fold, we aligned all the 67,994 features of the training set using sparse representation and empirically select the first 10,000 features in 200 intervals, and thus we had 50 (10,000/200) classification results in each fold. The overall classification accuracy was the average accuracy of classification with the same number of training data features across folds, and when the highest overall classification accuracy was got, the union of the selected features in each fold were regarded as the most discriminative features of 2.8 mm data. The corresponding 1.4 mm features of all the selected features in 2.8 mm data were defined as the original features (8 times the dimensionality of the selected 2.8 mm features) for the next sparse representation operation. The same operation was conducted in 1.4–0.7 mm data.

Given training data $X=\left[x_{1},x_{2},\cdots \;,x_{n}\right]\in {\mathbb{R}}^{d\times n}$ and the associated class labels ***y*** ∈ ℝ^*n*^, the sparse representation algorithm can be modeled as follows:

(1) $$\begin{array}{r} y=X^{T}w, \end{array}$$

where ***w*** ∈ ℝ^*d*^ is the weight vector to be solved and it should be as sparse as possible. It can be described as the following optimization problem:

(2) $$\begin{array}{r} \begin{array}{c} \min∥w{∥}_{0}\\ s.t.\;X^{T}w=y, \end{array} \end{array}$$

it is a ℓ_0_-norm problem which is difficult to get the solution although the solution is the most desirable to Equation 1.

Under practical conditions, the ℓ_0_-norm problem is equivalent or approximately equivalent to the ℓ_1_-norm problem. It is convex and thus can be easily optimized. Besides, the utility of ℓ_1_-norm makes ***w*** less sensitive to noise. Consequently, we can get ***w*** by solving the following problem:

(3) $$\begin{array}{r} \begin{array}{c} \min∥w{∥}_{1}\\ s.t.\;X^{T}w=y, \end{array} \end{array}$$

considering that the constraint condition ***X***^*T*^***w*** = ***y*** makes ***w*** sensitive to outliers of ***X***, we suggested a new equation:

(4) $$\begin{array}{r} \underset{w}{\min}f\left(w\right)=∥X^{T}w-y{∥}_{1}+\gamma ∥w{∥}_{1}, \end{array}$$

thus we can get the approximate solution of Equation 1, and make sparse representation more robust.

We find Equation 4 is a specific form of the Robust Feature Selection (RFS) algorithm proposed by Nie et al. (2010). The RFS is based on regression and ℓ_2, 1_-norm sparsity regularization. Unlike the traditional least square regression which uses the squared ℓ_2_-norm loss, RFS emphasizes joint ℓ_2, 1_-norm minimization on both loss function and regularization. Before introducing RFS method, we first present the definition of the ℓ_2, 1_-norm of a matrix.

For the matrix ***M*** ∈ *R*^*n*×*m*^, its ℓ_2, 1_-norm is defined as:

(5) $$\begin{array}{r} ∥M{∥}_{2,1}=\overset{n}{\underset{i=1}{\sum }}\sqrt{\overset{m}{\underset{j=1}{\sum }}m_{ij}^{2}}=\overset{n}{\underset{i=1}{\sum }}{∥m^{i}∥}_{2}, \end{array}$$

where ***m***^*i*^ is the *i*-th row of ***M***.

Given training data $\left\{x_{1},x_{2},\cdots \;,x_{n}\right\}\in {\mathbb{R}}^{d}$, the RFS algorithm employs the one-vs-rest binary coding scheme to encode the class labels. Denote the total number of classes as *c*. The label vector of training data ***x***_*i*_ is represented by $y_{i}\in {\left\{0,1\right\}}^{c\times 1}$, such that *y*_*i*_(*j*) = 1 if ***x***_*i*_ belongs to the *j*-th category and *y*_*i*_(*j*) = 0 otherwise. The associated class labels of all data points are $\left\{y_{1},y_{2},\cdots \;,y_{n}\right\}\in {\mathbb{R}}^{c}$. RFS optimizes the following robust loss function:

(6) $$\begin{array}{r} \underset{W}{\min}\overset{n}{\underset{i=1}{\sum }}{∥W^{T}x_{i}+b-y_{i}∥}_{2}, \end{array}$$

where ***W*** ∈ ℝ^*d*×*c*^ is the projection matrix and ***b*** ∈ ℝ^*c*^ is the bias vector.

For simplicity, the bias ***b*** can be absorbed into ***W*** when the constant value 1 is added as an additional dimension for each data ***x***_*i*_(1 ≤ *i* ≤ *n*). Thus, the problem becomes:

(7) $$\begin{array}{r} \underset{W}{\min}\overset{n}{\underset{i=1}{\sum }}{∥W^{T}x_{i}-y_{i}∥}_{2}. \end{array}$$

For the sake of feature selection, we will add a sparse regularizer. Essentially, the *i*-row vector of ***W*** corresponds to the transformation vector of the *i*-th feature in regression. It can also be regarded as a vector that measures the importance of the *i*-th feature. Considering the task of feature selection, we expect that the transformation matrix holds the sparsity property for feature selection. More concretely, we expect that only a small number of row vectors of ***W*** are non-zeros. As a result, the corresponding features are selected since these features are enough to regress the original data ***x***_*i*_ to its label vector ***y***_*i*_. When we employ the ℓ_2_-norm of each row vector as a metrix to measure its contribution in this regression, the sparsity property, i.e., a small number of row vectors that are non-zeros, indicates the following RFS objective function:

(8) $$\begin{array}{r} \underset{W}{\min}\overset{n}{\underset{i=1}{\sum }}{∥W^{T}x_{i}-y_{i}∥}_{2}+\gamma \overset{n}{\underset{i=1}{\sum }}{∥w^{i}∥}_{2}, \end{array}$$

where ***w***^*i*^ denotes the *i*-th row of ***W***. The parameter γ is to balance the regression loss and the influence of sparse regularizer, and it was set to be the default value 0.01 suggested by Nie et al. (2010) through a series of empirical studies.

Denote data matrix $X=\left[x_{1},x_{2},\cdots \;,x_{n}\right]\in {\mathbb{R}}^{d\times n}$ and label matrix $Y={\left[y_{1},y_{2},\cdots \;,y_{n}\right]}^{T}\in {\mathbb{R}}^{n\times c}$, the objective function becomes:

(9) $$\begin{array}{l} \underset{W}{\min}J(W)=\sum_{i=1}^{n}{\left\|W^{T}x_{i}-y_{i}\right\|}_{2}+\gamma \sum_{i=1}^{n}{\left\|w^{i}\right\|}_{2}\\ \;={\left\|X^{T}W-Y\right\|}_{2,1}+\gamma {\left\|W\right\|}_{2,1}. \end{array}$$

The ℓ_2, 1_-norm based loss function makes RFS robust to outliers in data points and the ℓ_2, 1_-norm regularization enables RFS to select features across all data points with joint sparsity. Though both terms of the objective function are non-smooth, the problem can be solved efficiently with the reweighted method, which has been proved to be convergent. More details about the RFS algorithm can be seen in Nie et al. (2010).

After obtaining the solution of ***W***, features are ranked according to the value of $∥w^{i}{∥}_{2}$. In other words, the larger value of $∥w^{i}{∥}_{2}$ denotes that the *i*-th feature are more important. The features with less importance are then discarded.

### 2.3. Classification and Cross Validation

In each of the 10-fold cross validation, 90% samples were regarded as the training set and the remaining 10% samples were served as the testing set. The classifier used in this study was linear support vector machine (SVM), whose goal is to find a decision function:

(10) $$y=h^{\prime }x+b,$$

by solving the following optimization problem:

(11) $$\begin{array}{l} \;\underset{h,\varepsilon }{\min}\frac{1}{2}h^{2}+C\sum_{i=1}^{N}\xi_{i}\\ \text{s}.\text{t}.\;y_{i}\left(h^{\prime }x_{i}+b\right)\geq 1-\xi_{i}, \end{array}$$

where ***h*** denotes the normal of the hyperplane, ***x***_*i*_ denotes the *i*-th training vector and *y*_*i*_ is its corresponding lebel, ξ_*i*_ is the misclassification errors of non-separable cases, and C is the empirical risk and model complexity which was set to be 1 in this study. Females were labeled as -1 and males were labeled as 1, and thus the classification threshold was 0. The classification accuracy and the area under curve (AUC) of the receiver operating characteristic (ROC) curve were used as the classification performance index, and 1,000 times of permutation tests and 1,000 times of bootstrap tests were conducted to access the overall statistical significance of the classification results. In the permutation test of each fold, gender labels were randomly permuted when gender features kept stable, and 1,000 AUC values were used to construct a null distribution and compare with AUC value of using true gender labels. In each bootstrap test, 90% of the training set were randomly chosen as new training set, and inspired by the back projection stage of Wang et al. (2012), the weight of voxels was defined as the absolute of ***h***, and detailed equation was as follows:

(12) $$g=abs\;h=\text{abs}\sum_{i=1}^{N}\alpha_{i}y_{i}x_{i},$$

where ***g*** denotes the weight vector of voxels, α_*i*_ is the *i*-th value of alpha coefficient vector ***α*** in SVM, and *N* is the number of subjects in the training set. The mean of ***g*** in 1,000 times of bootstrap tests was the final weight vector $\bar{g}$.

## 3. Results

### 3.1. Gender Classification Results: AUC and Accuracy

**Figure 2:**
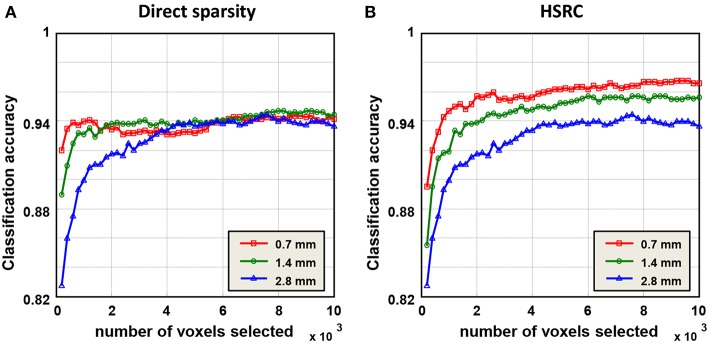
Classification results of the sparse representation, and the classification was a function of the number of voxels selected in each fold. In HSRC, the higher resolution data always have the higher classification accuracy, while in direct sparsity the classification accuracys of three resolution data are roughly the same. The highest accuracy is 96.77% which is got from 0.7 mm data using HSRC.

Results of gender classification using HSRC of three resolutions are provided in the top two rows of Table 1. The highest AUC and accuracy, both of which are got from 0.7 mm data, are 0.9925 and 96.77%, respectively. The relationship of classification accuracy and the number of selected features in each fold are provided in Figure 2B, which indicates that the classification accuracy of all the three resolutions improves rapidly up to 0.9 with a few voxels and with the same number of voxels, the higher resolution data always have higher classification accuracies with much less computation time (platform: Linux server with 2 Inter(R) Xeon(R) CUP @ 2.10 GHz, 28 kernels, 260 GiB Memory. CentOS 6.7, MATLAB R2015b, 1 fold RFS: 151.3 (0.7 mm) +158.8 (1.4 mm) +64.3 (2.8 mm) = 374.4 s for HSRC; 5682.6 s (0.7 mm) for direct sparsity) and storage demanded, but when direct sparsity is conducted in different resolution data, we do not see improvement of overall classification performance in higher resolution data, which proves that our HSRC algorithm indeed plays a part. The outcomes of conducting direct sparsity in different resolution data are in the median two rows of Table 1 and Figure 2A.

**Table 1:** AUC and accuracy for gender classification.

		**0.7 mm**	**1.4 mm**	**2.8 mm**
HSRC	AUC	0.9925	0.9868	0.9821
	Accuracy (%)	96.77	95.69	94.49
Direct sparsity	AUC	0.9829	0.9831	0.9821
	Accuracy (%)	94.34	94.70	94.49
PCA	AUC	0.9874	0.9870	0.9844
	Accuracy (%)	94.43	94.52	94.07

Gender classification using PCA was also conducted for comparing, and results are provided in the bottom two rows of Table 1, the classification performance of using PCA is comparable with using direct sparsity, but poor than using HSRC.

**Figure 3:**
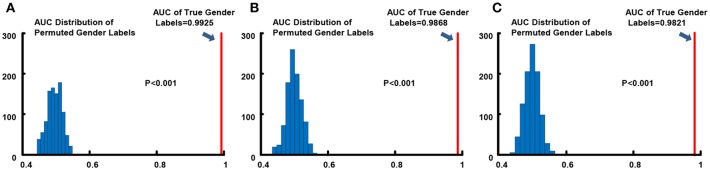
Permutation tests of AUC index for gender classification. **(A)** 0.7 mm; **(B)** 1.4 mm; **(C)** 2.8 mm. The light blue histograms indicates the null distributions of AUC for randomly permuted gender labels and the solid red line show the AUC when gender labels were true.

We conducted 1,000 times of permutation tests to testify the statistical significance of overall gender classification performance, and detailed results for all three resolution data are in Figure 3. Concurring with expectations, null distributions of the AUCs scattered around 0.5, which implied that the performance of the classifier for the randomly permuted data sets whose subjects were randomly labeled was just no better than the probability of getting positive side in random coin tossing. All of the AUC values for permuted labels fell behind the AUCs of real labels, which demonstrated high statistical significance of gender classification (*p* < 0.001) for all three resolutions.

### 3.2. Important 3-D Morphological Features in Gender Discrimination

**Figure 4:**
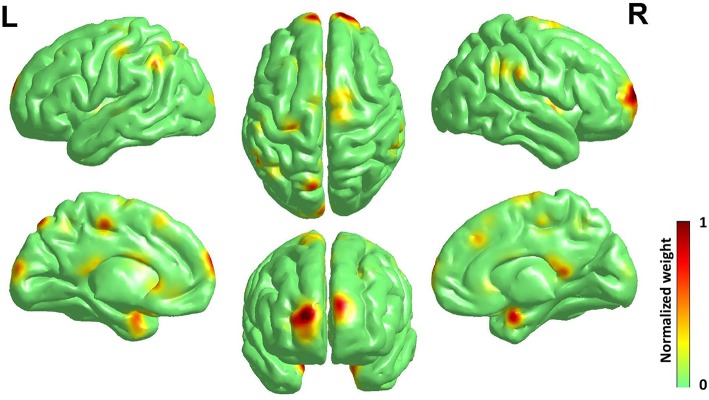
Surface rendering of discriminative regions of gender difference derived from normalized mean bootstrap result [visualized by BrainNet Viewer Xia et al., 2013]. The main morphology difference for gender exists mainly in the Frontal Lobe and the Limbic Lobe, others scattered in the Parietal Lobe, the Temporal Lobe, the Corpus Callosum, and the Precuneus.

As the best classification performance was obtained from 0.7 mm data, and other resolution data were downsampled from them, we conducted 1,000 times of bootstrap tests in 0.7 mm data, and the outcome is shown in Figure 4 and detailed information of the main clusters is in Table 2.

**Table 2:** The main locations of the voxels that were selected by HSRC in 0.7 mm.

**Cluster**	**Voxels**	**Hemisphere**	**MNI Coordinate**		
			**x**	**y**	**z**
Uncus	120	L	–25.5	2.1	–21.6
Uncus	143	R	20	4.2	–25.8
Superior Temporal Gyrus	103	L	–48.6	–23.8	2.9
Superior Frontal Gyrus	271	R	14.4	69.3	14.1
Corpus Callosum	141	R	2.5	–35	7.1
Superior Frontal Gyrus	139	L	–8.7	67.2	20.4
Middle Frontal Gyrus	117	L	–25.5	21.7	42.8
Precuneus	101	L	–9.4	–75.6	55.4

The main morphology difference for gender exists mainly in the frontal lobe and the limbic lobe, others scattered in the parietal lobe, the temporal lobe, the corpus callosum and the precuneus. Considering the high relevance of cortical 3-D morphology and GM, we compared our study and previous studies of gender difference with GM concentration, and found that our study had high accordance with the study of gender difference using T1w, T2w, and FA (Feis et al., 2013) and using GM concentration and fMRI (Wang et al., 2012), and also those using cortical thickness (Im et al., 2006; Sowell et al., 2007; Lv et al., 2010) in reporting the main gender difference in the frontal lobe, the limbic lobe, the parietal lobe and the temporal lobe. Moreover, there are reports of gender difference in the precuneus (Kaiser et al., 2008; Taki et al., 2011; Semrud-Clikeman et al., 2012) and the corpus callosum (Witelson, 1989; Allen et al., 1991; Bishop and Wahlsten, 1997).

### 3.3. Discriminative Ability of Brain Subregions

**Figure 5:**
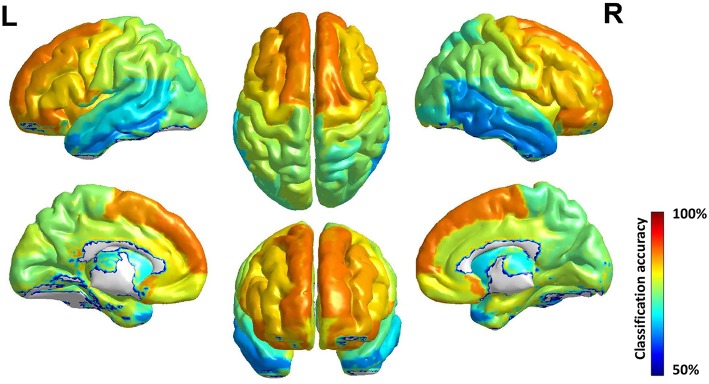
Surface rendering of gender classification accuracy of brain region in AAL. The discriminative ability of brain area improves roughly from behind to front, and the accuracy distribution of two hemi-spheres is roughly bilateral symmetrical.

The accuracy of each brain subregion in AAL for gender classification is in Figure 5, and the top and bottom 5 discriminative subregions and their classification accuracy are in Table 3. The most discriminative regions of gender exist in the front of the brain and the least discriminative regions are the temporal gyrus. It can be seen from Figure 5 that the accuracy distribution of two hemi-spheres is roughly bilateral symmetrical, which means that the corresponding brain areas of two hemi-spheres have approximately equal discriminative abilities in gender difference.

**Table 3:** The top and bottom 5 discriminative regions of AAL template and accuracy for gender classification, the highest gender classification accuracy distributed in the Frontal Lobe while the bottommost gender classification accuracy distributed in the Temporal Lobe.

**Top 5 discriminative regions [acc (%)]**	**Bottom 5 discriminative regions [acc (%)]**
Frontal_Sup_Medial_R (87.87)	Temporal_Inf_R (62.89)
Frontal_Sup_Medial_L (87.78)	Temporal_Pole_Mid_R (63.07)
Frontal_Sup_R (87.78)	Temporal_Inf_L (63.34)
Supp_Motor_Area_R (87.42)	Temporal_Pole_Mid_L (63.61)
Frontal_Sup_L (87.42)	Temporal_Mid_R (64.06)

An interesting phenomenon which should be paid attention to is that the brain subregions' discriminative ability for gender arises from posterior to anterior in the brain, and this phenomenon has high accordance with the evolution regular of human brain: these brain areas located in the anterior of the brain evolved first, while these posterior brain areas evolved later (Buckner and Krienen, 2013). A possible explanation is that these brain areas evolving advanced and better in human evolution history have more abundant and complex function, so they should develop first in individual brain to ensure the basic function, and with evolution the functional difference of gender grows thus the structural difference grows, too. And those brain areas evolving not so full have less functions and those functions are common among human beings.

The accuracies and AUCs of 7 RSN for gender classification are in Table 4. Considering the dimensionality of data, the classification of 7 RSN was conducted in 1.4 mm data. The most discriminative brain areas of gender difference mainly distribute in the default mode network, which is also indicated in Zhang et al. (2018). While a majority of the least discriminative regions belong to the visual network and dorsal attention network. The outcome offers a new evidence of the accordance between structural and functional brain.

**Table 4:** AUC and accuracy for gender classification of 7 RSN Networks.

**RSN network**	**1**	**2**	**3**	**4**	**5**	**6**	**7**
AUC	0.8782	0.9257	0.8849	0.9359	93.68	0.9285	0.9568
Accuracy (%)	80.05	86.16	81.22	86.25	86.88	85.44	90.21

*(1) Visual network; (2) Somatomotor network; (3) Dorsal attention network; (4) Ventral network; (5) Limbic network; (6) Frontoparietal contral network; (7) Default mode network*.

**Figure 6:**
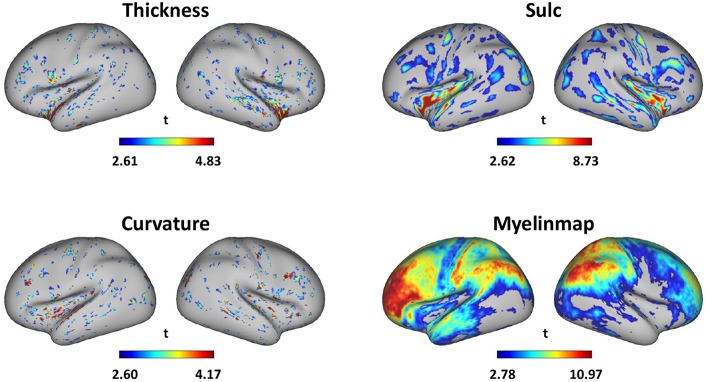
Gender difference of surface-based morphology denoted by absolute value of the *t*-value of two sample *t*-test (*p* < 0.01, visualized by workbench of the HCP).

Surface-based gender difference is in Figure 6 which shows that gender difference is most obvious in myelinmap of all the 4 surface-based morphology. The average gender classification accuracy in 10 times of 10-fold cross-validation of thickness, curvature, sulc and myelinmap are 0.8740, 0.8022, 0.8431, and 0.8820, respectively. The details of the most discriminative areas are as follows: isthmuscingulate, left superiortemporal, and right insula for cortical thickness; posteriorcingulate and insula for sulc; inferiorparietal, isthmuscingulate and left posteriorcingulate for curvtura; precuneus, rostralmiddlefrontal and superiorfrontal for myelinmap. Interestingly, myelinmap showed greater gender difference and those discriminative areas of myelinmap have high accordance with those areas we find in cortical 3-D morphology, especially in the frontal lobe and the precuneus; those discriminative areas in the other 3 surface-based morphology are mainly in the insula, which is also found in cortical 3-D morphology.

## 4. Discussion

In this study, we investigated gender difference of cortical 3-D morphology by proposing an HSRC approach, and got an accuracy of 96.77% in a 10-fold cross-validation. The robustness of classification was testified by permutation tests, and the frontal lobe was found the most discriminative region of gender difference in cortical 3-D morphology selected by HSRC. The superior frontal gyrus in AAL and the default mode network in RSN got the highest accuracy in template based classification. Moreover, the advantages of our proposed HSRC method were mentioned. Discussions are in the following.

There are reports of gender difference in cortical morphology (Im et al., 2006; Sowell et al., 2007; Lv et al., 2010; Sepehrband et al., 2018) and brain morphology changes in aging (Resnick et al., 2000; Bigler et al., 2002; Rusinek et al., 2003; Fjell et al., 2009) and multiple inherent brain disorders (Lieberman et al., 2001; Ashburner et al., 2003; Thompson et al., 2004; Jouvent et al., 2008; Aylward et al., 2010), and our proposed method may have the potential in auxiliary diagnosis of those disorders combined with other modalities. Theoretically brain morphology is less sensitive to the scan variables than GM concentration, which may help the fusion of sMRI data from different datasets, and thus our discovery may also offer a new thinking in dealing with multi-site MRI data (Ma et al., 2018; Yuan et al., 2018b; Zeng et al., 2018).

As far as we know, this work is the first to classify gender with original cortical 3-D morphology and to get an accuracy of over 95% in gender classification using morphological features. It encouraged us to draw a conclusion that genders can be distinguished on individual level by cortical 3-D morphology features, and supported those opinions in the aspect of brain morphology that males and females can be effectively classified (Chekroud et al., 2016; Rosenblatt, 2016; Anderson et al., 2018), as well as challenged these suggestions that brains are essentially indistinguishable in gender (Joel et al., 2015).

The result of bootstrap tests showed that those discriminative regions of gender difference found by cortical 3-D morphology had high accordance with those found by GM concentration and surface-based morphology in previous studies, especially in the frontal lobe, the limbic lobe and the partial lobe. We suggested a hypothesis that those gender difference of GM concentration, to some extent, may be the result of morphology difference.

Atlas-based morphology analysis indicated different discriminative abilities among brain areas, that is to say, some brain areas contributed much to the gender difference, while some areas exert a smaller influence, and even some areas had no contribution for gender difference, which may be referred to as so-called mosaic areas (Rippon et al., 2014; Joel et al., 2015). According to the brain areas classification results, those brain areas with complex functions and functions related to gender reap high accuracy in gender classification. The bootstrap results also show that the high difference voxels are located in the high difference brain areas, which is comprehensible and consistent with the classification results. Moreover, we found good symmetry in AAL-based morphology analysis which is rarely mentioned in previous studies of gender difference; RSN-based morphology analysis suggested that the default mode network is the most discriminative network, and the same result was also reported in the studies of gender difference using fMRI Zhang et al. (2018).

Considering that sample size was emphasized in recent studies (Ritchie et al., 2018), we particularly compared our findings with those using more than 1,000 samples (Chekroud et al., 2016; Gur and Gur, 2016; Anderson et al., 2018; Ritchie et al., 2018), and we found considerable accordance. First, the reported classification accuracies were more than 90% to support the opinions of sexual dimorphism with different MRI modalities. Second, the most discriminative areas/networks of gender difference were found to be the frontal lobe (Gur and Gur, 2016; Anderson et al., 2018; Ritchie et al., 2018) and the default mode network (Gur and Gur, 2016; Ritchie et al., 2018), further indicating high relevance of cortical morphology, GM concentration and fMRI based on large sample size.

The proposed HSRC algorithm was testified to be helpful in improving classification accuracy while reducing computation and storage resource for high-dimensional MRI data. It also selected features directly, making discriminative voxels more explainable in MRI data and may help to accurately locate lesion of diseased brain (Antel et al., 2003; Lladó et al., 2012).

We noticed several possible limitations in this work. Firstly, there are papers suggesting that important gender difference also exists in subcortical structures like cerebellum, amygdala and hippocampus (Giedd et al., 2012; Ruigrok et al., 2014). As cortical thickness of these subcortical structures is much less than that of the cerebral cortex, it cannot be automatically segmented by the pipelines offered by the HCP at present (Glasser et al., 2013). Since morphology data provided by the HCP did not include these subcortical structures so far, the influence of subcortical morphology to gender difference was not studied. Secondly, the effect of aging on brain morphology was not discussed because of narrow age range of adults (22–37 years old) in our study. Thirdly, because of the lack of T2w images, we have not conducted multi-site experiment to test the robustness of brain morphology by now. Moreover, although we have conducted dimension reduction, linear SVM and cross-validation to alleviate the risk of overfitting in the classification methodology as far as possible, an independent dataset is still required to validate the generalizability of our proposed model, which should be done once possible in the future.

## Ethics Statement

This study was carried out in accordance with the recommendations of “name of guidelines, name of committee” with written informed consent from all subjects. All subjects gave written informed consent in accordance with the Declaration of Helsinki. The protocol was approved by the “name of committee.”

## Author Contributions

DH designed the study. ZL conducted the experiment. ZL, CH, and LW wrote the article.

### Conflict of Interest Statement

The authors declare that the research was conducted in the absence of any commercial or financial relationships that could be construed as a potential conflict of interest.
