# Corrigendum: Gender Identification of Human Cortical 3-D Morphology Using Hierarchical Sparsity

**DOI:** 10.3389/fnhum.2019.00219

**Published:** 2019-06-28

**Authors:** Zhiguo Luo, Chenping Hou, Lubin Wang, Dewen Hu

**Affiliations:** ^1^College of Mechatronics and Automation, National University of Defense Technology, Changsha, China; ^2^College of Science, National University of Defense Technology, Changsha, China; ^3^Cognitive and Mental Health Research Center, Beijing Institute of Basic Medical Science, Beijing, China

**Keywords:** cortical three-dimensional morphology, gender difference, hierarchical sparse representation classifier, magnetic resonance imaging, multivariate pattern analysis

An author name was incorrectly spelled as “Chengping Hou.” The correct spelling is “Chenping Hou.”

The authors apologize for this error and state that this does not change the scientific conclusions of the article in any way. The original article has been updated.

